# Crosstalk between pluripotency factors and higher-order chromatin organization

**DOI:** 10.1080/19491034.2016.1248013

**Published:** 2016-10-19

**Authors:** Clara Lopes Novo, Peter Rugg-Gunn

**Affiliations:** Epigenetics Program, Babraham Institute, Cambridge, UK

**Keywords:** chromatin, development, embryonic stem cells, epigenetics, pericentromeric heterochromatin, pluripotency

## Abstract

Pluripotent cells are characterized by a globally open and accessible chromatin organization that is thought to contribute to cellular plasticity and developmental decision-making. We recently identified the pluripotency factor *Nanog* as a key regulator of this form of chromatin architecture in mouse embryonic stem cells. In particular, we demonstrated that the transcription factors *Nanog* and *Sall1* co-dependently mediate the epigenetic state of pericentromeric heterochromatin to reinforce a more open and accessible organization in pluripotent cells. Here, we summarize our main findings and place the work into a broader context. We explore how heterochromatin domains could be targets of transcriptional networks in pluripotent cells and are coordinated with cell state. We propose this integration may be to balance the requirement for a dynamic and plastic chromatin organization in pluripotent cells, together with priming for a more restrictive nuclear compartmentalization that is triggered rapidly upon lineage commitment.

## Introduction

Pluripotent cells, including embryonic stem cells (ESCs) and cells from early-stage mouse embryos, have a unique chromatin architecture that is characterized by an unusually open and decondensed genome.^1-3^ This higher-order nuclear organization differs drastically from the chromatin distribution that is observed in most other cell types, in which heterochromatin domains contain highly compacted chromatin that are readily discernable from euchromatin compartments.^4,5^ Remarkably, rapid compaction of the genome and reinforcement of heterochromatin silencing are changes that occur at the earliest stages during the transition from pluripotency to lineage commitment.^6,7^ Conversely, remodelling of the genome that culminates in an open and decompacted nuclear organization similar to ESCs occurs at a late stage during reprogramming and is required for the successful formation of induced pluripotent stem cells.^8,9^ Despite being a hallmark of pluripotency and thus fundamental to an in-depth understanding of stem cell and developmental biology, the molecular mechanisms that underpin these changes in nuclear organization are only partially defined. Additionally, why pluripotent cells acquire this open and dispersed chromatin configuration remains unknown. Here, we discuss our recent report that identified a role for the core pluripotency factor *Nanog* in controlling the nuclear architecture of mouse ESCs, thereby describing a new mechanism directly connecting cell state with higher-order genome organization.^10^

### *Nanog* is required for chromatin remodelling in pluripotent cells

Several reports had anticipated a crosstalk between the pluripotent regulatory network and chromatin organization in ESCs. For example, the genomic targets of the master pluripotent transcription factors (OCT4, SOX2 and NANOG) are co-occupied by proteins that directly control chromatin condensation, such as UTF1, or that promote chromatin looping, like the Cohesin and Mediator complexes, suggesting the potential for recruitment of chromatin remodellers by transcription factors.^7,11, 12^ Furthermore, the compaction of chromatin that is triggered as ESCs or cells from early-stage mouse embryos start to exit pluripotency occurs concurrently with the downregulation of *Nanog*, thus raising the possibility of a connection between NANOG levels and chromatin organization.^13-16^

To investigate the interplay between *Nanog* and chromatin organization, we analyzed chromatin architecture in a panel of pluripotent cell lines that were engineered to express *Nanog* at different levels.^17^ Applying electron spectroscopy imaging to quantify chromatin fiber density, we found that NANOG levels correlated with chromatin “openness” (i.e. reduced fiber density), particularly within heterochromatin compartments. We then measured the distribution and size of chromocentres by visualizing the heterochromatic marker histone 3 lysine 9 trimethylation (H3K9me3). We found that NANOG levels correlated with fewer, but larger chromocentres – a distinct organization that is highly characteristic of pluripotent cells. Additionally, *Nanog*-deficient ESCs had compacted and reorganized heterochromatin domains in a pattern similar to more developmentally mature cell types such as epiblast stem cells (EpiSCs), despite the ESCs remaining functionally undifferentiated. This exciting finding suggested that chromatin organization and the pluripotent state could be experimentally uncoupled, and therefore provide a tractable model to study connections between the 2 pathways. In particular, the requirement for *Nanog* to maintain an open and dispersed heterochromatin organization in pluripotent cells hinted that *Nanog* could be a “sought after” link for the anticipated crosstalk between master pluripotency factors and nuclear organization. We further tested this hypothesis using a more stringent assay by asking whether *Nanog* could trigger heterochromatin remodelling when ectopically expressed. We exploited the endogenous low levels of *Nanog* in EpiSCs and analyzed the distribution of chromatin before and after overexpression of NANOG. Notably, forced expression of NANOG in EpiSCs was sufficient to decondense heterochromatin and to redistribute chromocentres into fewer and larger foci, similar to the distribution observed in ESCs. Importantly, *Nanog* was the only pluripotency factor tested (others included Klf2, Klf4, Esrrb, Nr0b1, Prdm14, Dppa3, and Nr5a2) with the potential to remodel heterochromatin. Together, these results revealed a new function for *Nanog* in bridging the pluripotent state with the maintenance of an open and decondensed nuclear architecture.

### *Nanog*-dependent maintenance of open pericentromeric heterochromatin in ESCs

To better understand how *Nanog* contributes to genome architecture, we focused on the nuclear compartments that were most sensitive to *Nanog*-dependent remodelling: the pericentromeric heterochromatin (PCH). PCH are the main constituents of mouse heterochromatin and are formed from major satellite repeats that comprise 10% of the genome. PCH are essential for genetic stability and centromere formation. In most somatic cells, PCH are transcriptionally repressed with high levels of H3K9me3 and condensed chromatin fibers. Importantly, deletion of *Suv39h1/2* or *Dicer* in somatic cells leads to a reduction in the levels of repressive modifications within heterochromatin domains, to the transcriptional upregulation of PCH and to an increase in mitotic defects such as chromosome missegregation.^18,19^ In contrast, PCH of ESCs have lower relative levels of H3K9me3, are composed of open and decondensed fibers and are transcriptionally more active.^20,21^ Crucially, high endogenous levels of PCH transcription in ESCs is not associated with chromosome missegregation, suggesting that ESCs tolerate or even require a unique PCH identity.^19^ As we observed a *Nanog*-dependent reorganization of PCH compartments, we reasoned that *Nanog* could have a novel role in mediating the epigenetic state of PCH. Excitingly, we discovered that NANOG binds directly to major satellite DNA within PCH in ESCs and also when ectopically expressed in EpiSCs. Additionally, we found that in these cell types NANOG occupancy at major satellites had a positive impact on transcription and in preserving the open heterochromatic state of these regions through lower levels of H3K9me3 and SUV39H1. Interestingly, *Nanog* was unable to remodel PCH organization when expressed in fibroblast cells, suggesting that its function may be restricted to early embryo or stem cell types. A previous study showed that in mouse fibroblast cells the transcription factors PAX3 and PAX9 bind to PCH and contribute to the establishment of their epigenetic state.^22^ Together, this evidence leads us to suggest that cell-type specific regulatory networks participate in the establishment of distinct epigenetic states of PCH.

### A new complex for the regulation of pericentromeric heterochromatin in ESCs

At this point, it was unclear whether NANOG required additional regulators to mediate PCH identity and organization in ESCs. From the literature, the heterochromatin-binding transcriptional repressor SALL1 was a possible candidate as SALL1 is able to bind major satellite DNA and to also control transcriptional activation/repression in association with NANOG in ESCs.^23,24^ It was not known, however, if SALL1 has a functional role in regulating PCH in ESCs. We confirmed an interaction between NANOG and SALL1 by immunoprecipitation. We further demonstrated that SALL1 is a cofactor required for NANOG-mediated PCH remodelling, as deletion of *Sall1* mimics the *Nanog*-deficient ESC phenotype, and *Sall1* is also required for NANOG-induced PCH remodelling in EpiSCs. Importantly, deletion of either *Nanog* or *Sall1* induced misregulation of the ESC-specific PCH state and this occurred without loss of ESC identity as evaluated by transcriptional and immunofluorescent phenotyping. To determine which domains of NANOG are required for heterochromatin remodelling, we ectopically expressed several truncated NANOG proteins in EpiSCs and found that the strong C-terminal CD2 transactivator domain was necessary to remodel PCH organization. Using a genetic trick, we were also able to show that the NANOG CD2 domain was sufficient to initiate PCH remodelling in EpiSCs, and could do so in the absence of SALL1. This was accomplished by fusing NANOG–CD2 with a transcription activator-like effector (TALE) that targets major satellite DNA, thereby artificially recruiting NANOG–CD2 to PCH. In sum, these results led us to propose that an open PCH organization in ESCs is maintained co-dependently by NANOG and SALl1 through regulation of major satellites.

Our results suggest that the reduction of *Nanog* expression that occurs during ESC to EpiSC transition is sufficient to trigger changes in PCH identity and organization. As fluctuations in *Nanog* levels can lead to remodelling of higher-order nuclear organization, our findings raise important consequences for the long-term stability of ESCs and their specialized cell-types that are derived upon differentiation. Conversely, as heterochromatin decompaction and endogenous *Nanog* induction are critical events that occur at a late stage in cell reprogramming, it is possible that these two processes might be connected in other contexts. It will be interesting to determine whether an association between pluripotent state and genome organization, similar to our findings in ESCs, are conserved during cell reprogramming. We suggest that this future line of work could have important applications including the improved generation of a more stable reprogrammed pluripotent cell type through expression of factors (such as *Nanog*) that might be able to increase the efficiency of chromatin remodelling. Additionally, considering that disturbance of the transcriptional and epigenetic state of pericentromeric regions in somatic cells leads to mitotic errors and genomic instability, it remains of great interest to investigate whether there is any genetic impact as a result of PCH misregulation that is induced upon the deletion of *Nanog* or *Sall1* in ESCs. Finally, it is also important to investigate the mechanisms of heterochromatin regulation in ESCs. In somatic cells, major satellites are transcribed into long RNAs where the forward transcript remains localized in *cis* and can recruit HP1 to PCH. In turn, this is further stabilized by SUV39H1/2 and H3K9me3, which recruit more HP1 to create a positive reinforcement at PCH.^25^ The function of pericentromeric regions could, therefore, depend not just on their transcriptional state but also on the type of transcripts being formed. It would be interesting to further investigate whether the reduction of major satellite transcription observed in *Nanog*-deficient ESCs and in EpiSCs also affects their transcript length; in particular if long transcripts are generated that could initiate the accumulation of the heterochromatic marks that we observed in these cells.^10^

### The silencing of noncoding loci: A controlled requirement for pluripotency exit?

An intriguing aspect of pluripotency is its tolerance or even requirement for an open and active epigenetic state at heterochromatic loci. Positive regulation of major satellite transcription and decondensed heterochromatin is one of the direct outcomes of the pluripotency factor *Nanog*, interlinking pluripotency maintenance with an open and transcriptionally permissive PCH. *In vivo* evidence also supports a likely requirement for active pericentromeric transcription in early development, as two-cell stage mouse embryos display bursts of forward and reverse major satellite transcription that were shown to be essential for chromocentre formation and developmental progression of the embryo.^26^ In addition, *Dicer*-depleted mouse ESCs accumulate increased levels of major satellite transcripts and, while this does not seem to impact their undifferentiated state, the mutant cells do have an impaired differentiation potential as embryoid body-like structures form but fail to differentiate. Importantly, *Dicer*-depleted ESCs remain viable with no signs of genomic instability, thereby supporting our findings that ESCs tolerate and perhaps require an open and transcriptionally permissive PCH state to maintain pluripotency and viability.^19,27^

Strikingly, the tolerance or requirement for open and active constitutive heterochromatin in pluripotent cells does not seem to be exclusive to PCH. For example, similar to PCH, telomeres in ESCs and in induced pluripotent stem cells were shown to have a lower density of H3K9me3 and H4K20me3 compared to differentiated cells, resulting in a “relaxed” chromatin state proposed to facilitate telomerase access to telomeres for regulation of their length.^28^ Equally, most rDNA (rDNA) cassettes are active in mouse ESCs but approximately half undergo inactivation and condensation into heterochromatin in differentiated cells. The rDNA silencing mediated by pRNA was shown to be a crucial step for the exit from pluripotency, as removal of the silencer pRNA retains ESCs in a pluripotent state with a low number of heterochromatic loci.^29^ Finally, pluripotency-specific origins of replication have a curious epigenetic signature that are enriched for pluripotency transcription factor binding sites, such as NANOG and OCT4.^30^

**Figure 1.**
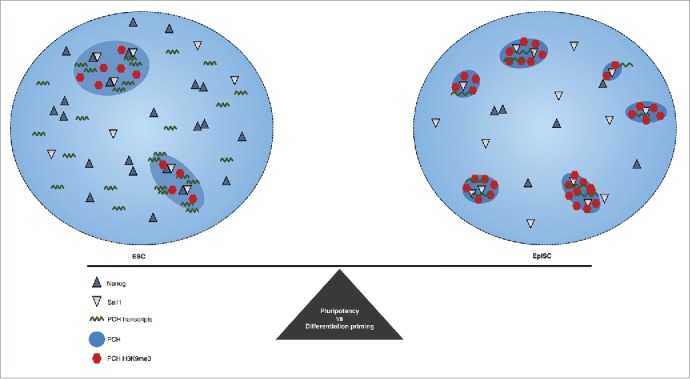
A model illustrating the importance of epigenetic regulation of noncoding PCH in mediating the balance between pluripotency and priming for differentiation. The pluripotent transcription factors NANOG and SALL1 co-bind to PCH to maintain an open heterochromatin state in ESCs that is characterized by elevated PCH transcript levels and lower levels of repressive marks including H3K9me3. As *Nanog* levels decrease upon transition to EpiSCs, PCH loci adopt an inaccessible and transcriptionally repressed epigenetic state, triggering the formation of constitutive heterochromatic and potentially repressive nuclear compartments.

Taken together, these findings pose an exciting model whereby the epigenetic maintenance of open and dynamic chromatin at constitutive heterochromatin contributes to the maintenance of pluripotency and is tightly regulated by the pluripotency network (Fig. 1). Subsequently, disassembly of the regulatory network including the downregulation of *Nanog* could trigger the silencing of constitutive heterochromatic loci (such as PCH, telomeres, rDNA) and expansion of heterochromatin compartments. Interestingly, heterochromatin compartments form transcriptionally repressive environments^31^ in differentiated cells but not in ESCs.^32^ Thus, the formation of repressive heterochromatin compartments during the exit of pluripotency could contribute to or accelerate the transcriptional repression of single copy regions. Alternatively, rather than directly driving nuclear organization, the stochastic “closeness” of several loci, including PCH, followed by a preferential co-segregation of heterochromatic regions, could form the basis for inactive nuclear compartments where repression could be stably maintained.^32^

## Conclusion

In conclusion, our work unveiled an important mechanism for controlling higher-order nuclear organization in mouse ESCs. By assigning new roles to two well-known stem cell factors, we propose that *Nanog* and *Sall1* bridge the pluripotent state with the open and decompacted chromatin architecture that defines pluripotent cells. This unexpected control of PCH chromatin state could contribute to the understanding of epigenetic regulation of other processes important for genome function. Finally, by suggesting that the regulation of heterochromatin is tightly controlled by the pluripotency network, we strengthen a potential role for noncoding loci in the modulation of higher-order chromatin organization during development and stem cell differentiation.
